# Rapid evolution of symbiont‐mediated resistance compromises biological control of aphids by parasitoids

**DOI:** 10.1111/eva.12532

**Published:** 2017-09-03

**Authors:** Heidi Käch, Hugo Mathé‐Hubert, Alice B. Dennis, Christoph Vorburger

**Affiliations:** ^1^ Aquatic Ecology Eawag Dübendorf Switzerland; ^2^ Institute of Integrative Biology ETH Zürich Zürich Switzerland; ^3^ Institute for Biochemistry & Biology University of Potsdam Potsdam Germany

**Keywords:** aphids, *Aphis fabae*, biological control, defensive symbiosis, *Hamiltonella defensa*, *Lysiphlebus fabarum*, parasitoid, resistance

## Abstract

There is growing interest in biological control as a sustainable and environmentally friendly way to control pest insects. Aphids are among the most detrimental agricultural pests worldwide, and parasitoid wasps are frequently employed for their control. The use of asexual parasitoids may improve the effectiveness of biological control because only females kill hosts and because asexual populations have a higher growth rate than sexuals. However, asexuals may have a reduced capacity to track evolutionary change in their host populations. We used a factorial experiment to compare the ability of sexual and asexual populations of the parasitoid *Lysiphlebus fabarum* to control caged populations of black bean aphids (*Aphis fabae*) of high and low clonal diversity. The aphids came from a natural population, and one‐third of the aphid clones harbored *Hamiltonella defensa*, a heritable bacterial endosymbiont that increases resistance to parasitoids. We followed aphid and parasitoid population dynamics for 3 months but found no evidence that the reproductive mode of parasitoids affected their effectiveness as biocontrol agents, independent of host clonal diversity. Parasitoids failed to control aphids in most cases, because their introduction resulted in strong selection for clones protected by *H. defensa*. The increasingly resistant aphid populations escaped control by parasitoids, and we even observed parasitoid extinctions in many cages. The rapid evolution of symbiont‐conferred resistance in turn imposed selection on parasitoids. In cages where asexual parasitoids persisted until the end of the experiment, they became dominated by a single genotype able to overcome the protection provided by *H. defensa*. Thus, there was evidence for parasitoid counteradaptation, but it was generally too slow for parasitoids to regain control over aphid populations. It appears that when pest aphids possess defensive symbionts, the presence of parasitoid genotypes able to overcome symbiont‐conferred resistance is more important for biocontrol success than their reproductive mode.

## 1 INTRODUCTION

Growing public concern about the use of chemical products in food production and the frequent evolution of resistance to pesticides is leading to an increased adoption of biological control as a sustainable way to reduce damage by pest insects (Heimpel & Mills, 2017; van Lenteren, 2012). On a global scale, aphids are among the most important agricultural pests (Dedryver, Le Ralec, & Fabre, 2010), and they are notorious for evolving insecticide resistance (Foster, Devine, & Devonshire, 2007). Among the many natural enemies aphids have, parasitoid wasps of the subfamily Aphidiinae (Hymenoptera: Braconidae) are particularly useful as biocontrol agents because of their short generation time and high fecundity, and because they prey exclusively on aphids (Powell & Pell, 2007). In most species, females can produce more than 200 eggs (Starý, 1970), which they inject singly into aphids. The parasitoid larvae develop inside the still active aphid until they kill their hosts and pupate within a cocoon inside the host's husk, which is referred to as a mummy. At favorable temperatures (≥20°C), the new generation of adult wasps hatches within just 2 weeks of oviposition.

Due to these desirable attributes, aphidiine parasitoids have been used in classical importation biological control, but their inundative release in field crops is still economically inviable compared to insecticide treatments, because of the large number of individuals required and their rapid loss from treated fields by dispersal (Boivin, Hance, & Brodeur, 2012). In greenhouse crops, on the other hand, the active release of parasitoids is successful and widely adopted (van Lenteren, 2012; Powell & Pell, 2007). A growing industry of commercial breeders supplies aphidiine parasitoids as biocontrol agents.

Although they are not being produced commercially against aphids at the moment, asexual, all‐female parasitoids are particularly promising for biological control (Stouthamer, 1993). Thelytoky, the production of diploid female daughters from unfertilized eggs, occurs in several taxa of the aphidiine genus *Lysiphlebus* (Belshaw, Quicke, Völkl, & Godfray, 1999; Petrović et al., 2015; Sandrock, Schirrmeister, & Vorburger, 2011). Thelytokous parasitoids could be more effective as biocontrol agents because only females kill hosts and because – all else being equal – asexuals have a twofold reproductive advantage and thus a higher population growth rate than sexuals (the twofold cost of males, Maynard Smith, 1978). In reality, the cost of sex is likely to be less than twofold in aphidiines, because under haplo‐diploid sexual reproduction (arrhenotoky), females have control over the sex ratio of their offspring via the fertilization of eggs with stored sperm, and most aphidiine parasitoids tend to produce female‐biased sex ratios (e.g., Chau & Mackauer, 2000; Kant, Minor, & Trewick, 2012; Mackauer & Völkl, 2002). Nevertheless, thelytokous parasitoids should have a substantial reproductive advantage over arrhenotokous parasitoids.

A potential disadvantage of thelytoky from a biocontrol perspective is the reduced evolutionary potential of asexual lines. This may be important because aphid populations can respond rapidly to selection by parasitoids (Herzog, Müller, & Vorburger, 2007), which is especially problematic when some aphid clones harbor heritable defensive endosymbionts such as *Hamiltonella defensa*,* Serratia symbiotica*,* Regiella insecticola,* or the X‐type symbiont (Oliver, Russell, Moran, & Hunter, 2003; Guay, Boudreault, Michaud, & Cloutier, 2009; Vorburger, Gehrer, & Rodriguez, 2010; reviewed in Vorburger, 2017). One of these symbionts, the gammaproteobacterium *Hamiltonella defensa* (Moran, Russell, Koga, & Fukatsu, 2005), increases aphid resistance to parasitoid wasps particularly strongly and consistently (Oliver, Moran, & Hunter, 2005; Oliver et al., 2003; Schmid, Sieber, Zimmermann, & Vorburger, 2012; Vorburger, Sandrock, Gouskov, Castañeda, & Ferrari, 2009). Laboratory cage experiments have shown that the prevalence of infection with *H. defensa* can increase rapidly in response to selection by parasitoids (Oliver, Campos, Moran, & Hunter, 2008; Sanders et al., 2016), leading to increased population resistance. Aphid parasitoids, in turn, are also able to adapt to the presence of defensive symbionts in their host populations (Dion, Zele, Simon, & Outreman, 2011; Rouchet & Vorburger, 2014), but only if they possess the necessary genetic variation. In *Lysiphlebus fabarum*, thelytoky is under genetic control (Sandrock & Vorburger, 2011) and known to occur via a mechanism termed central fusion automixis (Belshaw & Quicke, 2003). Thelytokous lines lose heterozygosity over time and become genetically homogeneous, virtually like clones (Vorburger, 2014). Asexual parasitoids may thus lack the ability to coevolve with their hosts and adapt to the evolution of increased resistance. As sexual and asexual lines of *L. fabarum* are very closely related (Sandrock et al., 2011), they are generally comparable in terms of generation time and fecundity (Engelstädter, Sandrock, & Vorburger, 2011). That said, a recent study reported small but significant differences in life‐history traits (e.g., slower development but higher egg number in asexuals), although based on comparing just a single line each (Ameri, Rasekh, & Mohammadi, 2015).

Whether the faster population growth rate of asexuals or the higher evolutionary potential of sexuals is more advantageous at the time scale of typical biocontrol interventions is unknown. We tried to address this question in experimental populations of aphids and parasitoids, using a factorial design. Aphid populations with either high or low genotypic diversity were exposed to either sexual or asexual parasitoids. Aphid populations were partially infected with *H. defensa*. One‐third of the genotypes in both diversity treatments carried the symbiont, which corresponds closely to the frequency of infection in the natural population these genotypes were collected from. We followed aphid and parasitoid population dynamics to assess the effectiveness of biological control, and we tracked the genotypic composition to document (co‐)evolution.

## 2 METHODS

### 2.1 Insects

Our experiment employed 15 different clones of the black bean aphid, *Aphis fabae*, an important pest of broad bean (*Vicia faba*) and sugar beet (*Beta vulgaris*). All clones were established in the laboratory from single parthenogenetic females collected in the field in the vicinity of Zurich, Switzerland. One clone was collected in summer 2006, the others during summer 2012. Since their collection, they were maintained in the laboratory on broad beans at approx. 20°C and with a 16‐hr photoperiod, conditions that ensure continued parthenogenetic reproduction. All clones were genotyped at eight microsatellite loci (Coeur d'Acier, Sembene, Audiot, & Rasplus, 2004) to confirm that they represent distinct genotypes. They were further tested for infection with three facultative bacterial endosymbionts, *Hamiltonella defensa*,* Regiella insecticola,* and *Serratia symbiotica* (Moran et al., 2005), using diagnostic PCR with primers and cycling conditions as described in Ferrari, West, Via, and Godfray (2012). Five clones carried a heritable infection with *H. defensa,* and two clones carried a heritable infection with *R. insecticola*. *Hamiltonella defensa* increases *A. fabae*'s resistance to its most important parasitoid *L. fabarum* (Schmid et al., 2012), whereas *R. insecticola* does not appear to influence resistance to these parasitoids in *A. fabae* (Vorburger et al., 2009). We did not test for the X‐type symbiont because in a previous study, no infections with this symbiont were detected in a sample of over 400 *A. fabae* from Central Europe (Vorburger & Rouchet, 2016). Collection details and microsatellite genotypes of all aphid clones used in the experiments are provided in Table S1.

As parasitoids, we used a diverse sexual stock as well as five asexual lines of *L. fabarum*. The sexual stock was a mixture of nine accessions of sexual *L. fabarum* collected in June and September 2012 at six sites across Switzerland (Table S2). They were first kept separately for approx. 15 generations at large population sizes (≥50 individuals transferred per generation), reared on an *H. defensa*‐free clone of *A. fabae* that was different from those in the experiment. Two generations before the start of the experiment, wasps from all nine populations were pooled (20 females and approx. 10 males from each line) to produce a common, genetically variable stock. The five asexual lines of *L. fabarum* were started from single thelytokous females collected in the vicinity of Zürich either in 2006 (one line) or 2012 (four lines). We genotyped the asexual lines with microsatellites as in Sandrock, Frauenfelder, Von Burg, and Vorburger (2007) to verify that they represent distinct lines. Their genotypes and collection details are provided in Table S3.

### 2.2 Population cage experiment

The population cage experiment followed a factorial design, in which host populations of either high genotypic diversity (15 aphid clones) or low genotypic diversity (three aphid clones) were exposed to sexual or asexual parasitoids, with five replicate cages per treatment combination (20 cages in total). The high genotypic diversity cages contained all 15 aphid clones, of which five were infected with *H. defensa*; the low genotypic diversity cages each contained a subset of one *H. defensa*‐infected clone and two *H. defensa*‐free clones. Clones were assembled randomly into five subsets such that all 15 clones were used in the low genotypic diversity treatments (sampling without replacement). Each subset was represented once in combination with sexual parasitoids and once in combination with asexual parasitoids. Table S1 details which clones were used in which low genotypic diversity cages.

The experiment was started by placing 14 potted, 3‐week‐old broad bean plants into each cage (47.5 × 47.5 × 47.5 cm; BugDorm‐44545F; MegaView Science, Taiwan) and inoculating the plants with 150 adult aphids, that is, 10 aphids per clone for high genotypic diversity cages and 50 per clone for low genotypic diversity cages. Cages were placed in a climatized room with a 16‐h photoperiod at 22°C. Two weeks later, when the aphids had established sizeable populations of approx. 10,000–20,000 individuals of all life stages, parasitoids were introduced. For the asexual parasitoid treatment, we added four females from each of the five thelytokous lines per cage, that is, 20 females in total, and for the sexual parasitoid treatment, we added 20 presumably mated females together with 3–8 males from the mixed sexual stock. The experiment was then maintained for 13 weeks (approx. 10–11 aphid and 6–8 parasitoid generations). Twice a week we replaced two old potted plants with fresh plants. The old plants were cut at ground level and left in the cage for a week to allow aphids to move to other plants and wasps to hatch from mummies. Because it was impossible to fully count the large insect populations that established in the cages, we relied on sentinel plants to obtain a proxy of aphid and parasitoid population sizes. Weekly, one additional pot with two small, 2‐week‐old bean plants was added to each cage. The first of these plants served the estimation of aphid density and was cut and removed again after 1 week. We measured the total stem length of the plant and counted the live aphids on it (all developmental stages). Aphid density was then expressed as the number of aphids per cm stem length to account for differences in plant size. The second sentinel plant served the estimation of parasitoid density. It was harvested after 2 weeks because parasitoid development from oviposition to mummification takes approximately 9–10 days. We measured plant stem length and counted all mummies (hatched and unhatched) to express parasitoid density as the number of mummies per cm stem length. We did not count the live aphids on the second sentinel plant because in some cases plant condition started to deteriorate after 2 weeks, such that aphids started to emigrate, whereas the attached mummies remained a reliable indicator of parasitoid density.

The development of aphid and parasitoid populations was analyzed with a generalized linear mixed model in the statistical software R v. 3.3.2 (R Core Team, 2016), using the contributed package glmmADMB v.0.8.0 (Fournier et al., 2012). The numbers of aphids or parasitoid mummies per cm of plant stem length were fitted using a negative binomial distribution with log link, testing for the effects of host genotypic diversity, parasitoid reproductive mode, and count (i.e., week after the start of the experiment), as well as their interactions. Cage was included in the models as a random effect to account for the nonindependence of repeated counts from the same cages.

A time‐lagged, negative correlation between the change in parasitoid density and the change in host density would be a signature of parasitism affecting host population growth. Therefore, we used a linear mixed model to predict the change in estimated aphid density between two counts in all cages by the change in estimated parasitoid density in the same time interval as well as the change in parasitoid density in the two previous time intervals (1‐week lag and 2‐week lag), again including cage as a random effect.

### 2.3 Genotypic composition of experimental populations

Selection by parasitoids may lead to changes in the clonal composition of aphid populations (e.g., Herzog et al., 2007). To track aphid genotype frequencies, we collected and genotyped a haphazard sample of aphids from each cage 5 weeks after parasitoid addition (midpoint) and at the end of the experiment (if aphid populations persisted). Samples consisted of 30 individuals for the low genotypic diversity treatment and of 50 individuals for the high genotypic diversity treatment. DNA of individual aphids was prepared using a Chelex protocol (see Vorburger, Siegrist, & Rhyner, 2017) and genotyped at eight microsatellite loci described in Coeur d'Acier et al. (2004). For the 10 cages with asexual parasitoids, we also documented the changes in the relative frequencies of the five asexual lines originally introduced. For the midpoint sample, only a small proportion of the available wasps was haphazardly collected to minimize the influence on parasitism pressure (mean sample size 32 ± 12.5 *SD*). For the endpoint, seven plants per cage were cut and sealed in cellophane bags to collect all parasitoids that emerged from the mummies on these plants. DNA was also prepared with Chelex, and the wasps were genotyped at a subset of the microsatellite loci described in Sandrock et al. (2007) that distinguished the five asexual lines. The sexual parasitoids were not genotyped because a small number of presumably neutral microsatellite markers is unlikely to yield any information on selection in sexual populations.

### 2.4 Follow‐up experiment: Infection matrix

The strong changes we observed in the genotypic composition of experimental populations prompted us to do a follow‐up experiment to estimate the susceptibility of all 15 aphid clones to the five asexual lines as well as the sexual population of *L. fabarum*. Every aphid clone was exposed to every wasp line (90 combinations), and we did three replicate exposures per combination in three complete randomized blocks that were carried out over three consecutive days. We first split up each aphid clone into the required number of replicates, assigned them to random positions within each block, and reared the aphids for one generation on small potted plants covered with cages. This first generation of rearing was required to render replicates truly independent. Exposing them directly to wasps would have risked that environmental maternal effects carried over from the stock could have influenced our estimates. The second aphid generation was started by placing three adult aphids from each replicate colony on a new plant, where they reproduced for 24 hr before getting discarded. Two days later, the similar‐aged (48‐ to 72‐hr‐old) cohorts of offspring were counted and exposed to single female wasps for 24 hr. Ten days after exposure to wasps, all successfully parasitized aphids were recognizable as mummies. The number of mummies, surviving aphids, and aphids that were found dead (but not parasitized) was recorded. Aphids that could not be recovered were assumed to be dead.

We took the proportion of mummies among all aphids initially exposed to parasitoids as our estimate of susceptibility to parasitism. These proportions were arcsin square‐root transformed and analyzed with a linear mixed model (R package lme4; Bates, Maechler, Bolker, & Walker, 2015), testing for the fixed effects of *H. defensa* infection, parasitoid line, and their interaction, as well as for the random effects of block, aphid clone (nested within *H. defensa* infection), and the aphid clone × parasitoid line interaction. *p* Values for the fixed effects were calculated using *F* tests with Satterthwaite's approximation, and *p* values for the random effects were calculated based on likelihood ratio chi‐square tests using the lmerTest library in R (Kuznetsova, Brockhoff, and Christensen, 2015).

## 3 RESULTS

### 3.1 Population dynamics: Protected aphids escape control by parasitoids

**Figure 1:**
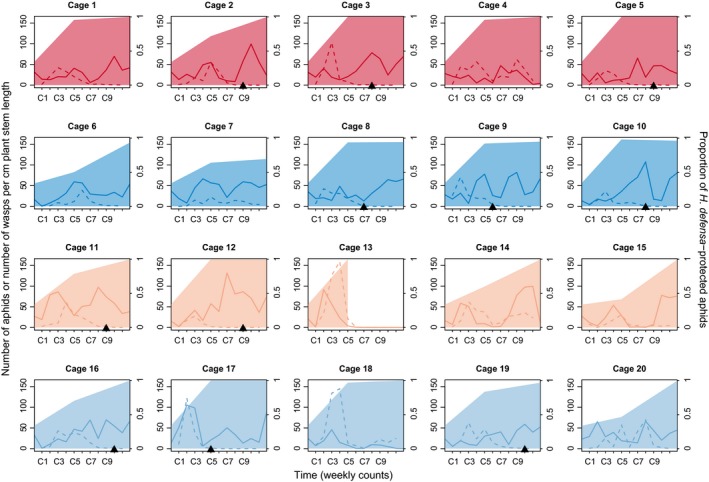
Aphid and parasitoid wasp population dynamics. Development of aphid and wasp population size in 20 experimental cages estimated by weekly counts of aphids (solid line, count 0 to count 12) and parasitoid mummies (dashed line, count 1 to count 11) on sentinel plants. The number of individuals was normalized by sentinel plant stem length, and the number of mummies was multiplied tenfold to increase visibility. Shaded areas represent the estimated proportion of aphids infected with the resistance‐conferring endosymbiont *Hamiltonella defensa*. Treatments are color coded: Red* *=* *asexual wasps, high aphid diversity (cages 1–5); blue* *=* *sexual wasps, high aphid diversity (cages 6–10); orange* *=* *asexual wasps, low aphid diversity (cages 11–15); light blue* *=* *sexual wasps, low aphid diversity (cages 16–20). Black triangles on the *x*‐axis represent parasitoid extinctions

Parasitoids failed to control aphid populations in the majority of cages, which resulted in large aphid populations at the end of the experiment (Figure 1). In five of these cages, only very few parasitoids persisted, and in 11 cages, the parasitoids even went extinct before the end of the experiment. Exceptions included cage 13, where parasitoids controlled and finally extirpated aphids successfully, as well as cages 4, 14, and 18, where parasitoids persisted at reasonably high densities and where aphid densities were low or at least declining at the end of the experiment (Figure 1). These exceptions from the general outcome were spread over three different treatment combinations. Accordingly, the analysis of aphid and parasitoid counts showed that their densities varied significantly over time, but did not differ significantly between cages with high or low host genotypic diversity, nor between cages with sexual or asexual parasitoids. The interactions between any of these effects were not significant (Table 1).

**Table 1:** Results of a generalized linear model testing for treatment effects and count number on estimates of aphid and parasitoid densities in experimental cages. The model was fitted with glmmADMB (Fournier et al., 2012), using a negative binomial distribution and including cage as a random effect

Effect	Aphids			Parasitoids		
	*df*	LR χ^2^	*p*	*df*	LR χ^2^	*p*
Host genotypic diversity	1	0.16	.689	1	0.14	.712
Parasitoid reproductive mode	1	0.08	.777	1	1.26	.262
Count	12	24.76	.016	10	92.21	<.001
Host genot. div. × paras. repr. mode	1	0.64	.424	1	0.44	.506
Host genot. div. × count	12	7.84	.798	10	11.08	.351
Paras. repr. mode × count	12	14.22	.287	10	4.76	.907
Host genot. div. × paras. repr. mode × count	12	8.08	.779	10	3.70	.960

Importantly, the lack of aphid control in the majority of cages was not due to an initial failure of parasitoids to establish from the small inoculum we added. Virtually all cages showed an initial surge of parasitoid numbers, followed by their decline in most cages (Figure 1). In fact, a negative relationship between changes in parasitoid density and changes in aphid density with a 2‐week delay suggests that parasitoids did have some effect on aphid populations (Table 2). However, this effect seems to be restricted to the early phase of the experiment. When we arbitrarily split the experiment at count 5, when the midpoint samples were taken, we see significant negative effects of changes in parasitoid density with a 1‐ and 2‐week delay on changes in aphid density during the early phase, but not during the later phase after count 5 (Table 2). In the later phase, there is a significant positive relationship between changes in parasitoid density and the simultaneous changes in aphid density (Table 2). We suspect that in the later phase of the experiment, when aphid populations were highly resistant and only very few mummies still formed, it was more likely to find any mummies on sentinel plants with larger aphid populations, thus explaining this positive relationship.

**Table 2:** Results of linear mixed models analyzing the effects of changes in parasitoid density with different time lags on changes in aphid density in the population cage experiment. Cage was included as a random effect

Time period	Effect (change in parasitoid density)	Estimate (*SE*)	*t‐*Value (*df*)	*p*
Whole experiment	Same week	0.504 (0.774)	0.652 (176)	.515
	Previous week	−0.851 (0.697)	−1.221 (176)	.224
	Two weeks before	−1.619 (0.711)	−2.276 (176)	.024
Early phase (up to count 5)	Same week	−0.975 (0.852)	−1.144 (56)	.257
	Previous week	−2.274 (0.922)	−2.467 (56)	.017
	Two weeks before	−3.387 (1.223)	−2.769 (56)	.008
Late phase (counts 6 – 12)	Same week	4.393 (1.896)	2.317 (116)	.022
	Previous week	0.271 (1.111)	0.244 (116)	.808
	Two weeks before	−1.184 (0.990)	−1.196 (116)	.234

### 3.2 Changes in aphid genotypic composition

**Figure 2:**
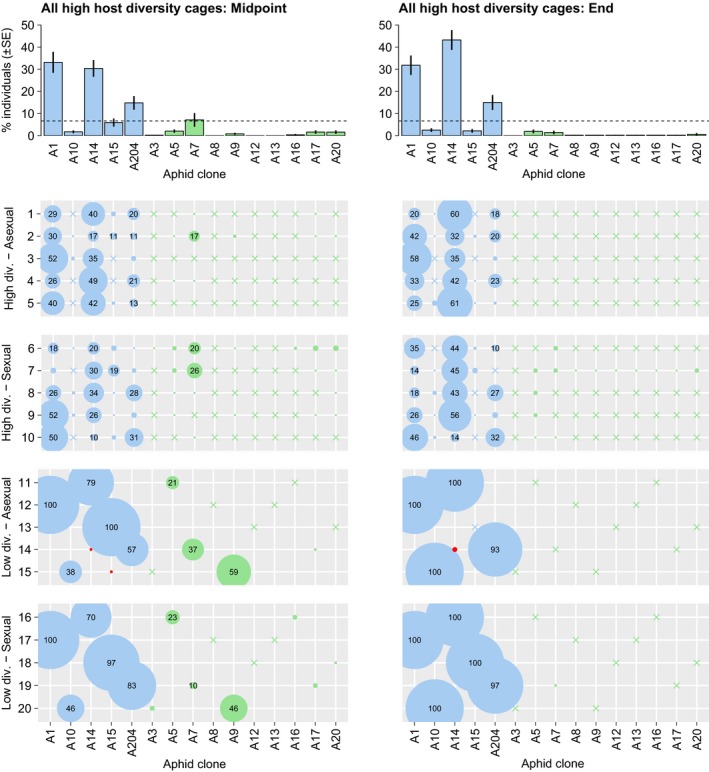
Parasitoids select for symbiont‐protected aphids. Bubble plots depicting the relative frequencies (in %) of *Hamiltonella defensa*‐infected (blue) and *H. defensa*‐free (green) aphid clones in subsamples from all experimental cages taken at the midpoint (left) and at the end of the experiment (right). Bars above summarize the mean frequencies of all cages with high host genotypic diversity (± *SE*). Red circles represent the rare cases of unexpected genotypes detected in three subsamples (only four individuals in total)

Genotyping of aphid subsamples revealed strong selection for *H. defensa*‐protected clones (Figure 2). All cages started with one‐third of the individuals belonging to clones infected with *H. defensa*. Over the first weeks, when parasitoid populations picked up, this proportion increased steeply in all cages, in some cases approaching or even reaching 100% in the midpoint samples at count 5 (Figure 1). At the end of the experiment, all 19 cages still containing aphids were dominated by clones possessing *H. defensa* (Figures 1 and 2). In cages with low genotypic diversity, this meant that populations became virtually monoclonal, with the single protected clone surviving. In cages with high genotypic diversity, genotyping showed that not all *H. defensa*‐protected clones fared equally well. Clones A14, A1, and A204 were consistently more successful than A10 and A15, which had become rare at the end of the experiment (Figure 2).

The genotyping of aphids also revealed very few unexpected genotypes in three cages (red points in Figure 2), suggesting that cross‐contamination between cages during the handling of the experiment could not be avoided completely. However, these were so rare that they cannot have influenced the outcome of the experiment (1 of 30 aphids in two cages at midpoint; 2 of 29 aphids in one cage at the end; no unexpected genotypes in any other cages).

### 3.3 Infection matrix

**Figure 3:**
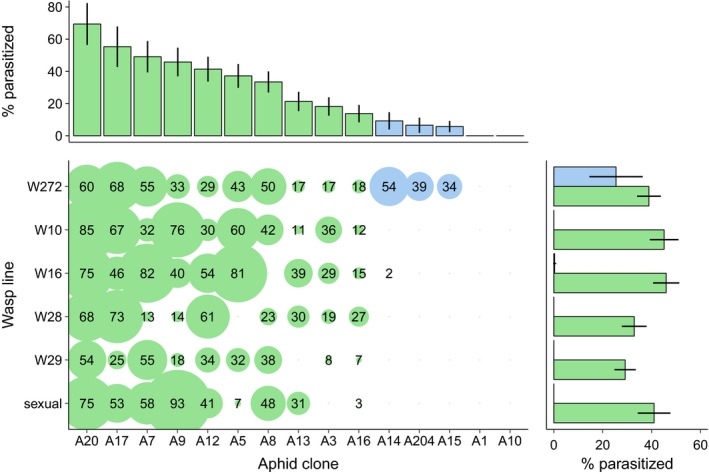
Results of the infection matrix experiment. Bubbles depict the mean percentage of successfully parasitized aphids from three replicate assays for all possible combinations of aphid clones and parasitoid lines used in the population cage experiment. Bars above show the average susceptibilities of aphid clones over all parasitoid lines (± *SE*), bars to the right show the average infectivities of the wasp lines across aphid clones, separately for *Hamiltonella defensa*‐protected (blue) and *H. defensa‐*free clones (green)

As expected, aphids infected with *H. defensa* were much more resistant to the parasitoids than aphids without this symbiont (Table 3, Figure 3), but there was also significant variation in susceptibility to parasitoids among aphid clones within these two groups. The six parasitoid lines differed somewhat in their average infectivity (Figure 3), yet this variation was not statistically significant. Interestingly, the *H. defensa*‐infected clones showed virtually complete resistance against all parasitoids except for the asexual line W272. This line was able to parasitize three of the five *H. defensa*‐protected clones (A14, A15, and A204), achieving rates of parasitism between 34% and 54% (Figure 3). The unique ability of line W272 to exploit aphids with *H. defensa* was not reflected in any statistically significant interactions (Table 2), presumably because of insufficient power with only three replicates per aphid clone‐parasitoid line combination, but the outcome of the cage experiment implies that this ability is biologically significant.

**Table 3:** Results of a linear mixed effects model on the proportion of aphids mummified by parasitoids in the infection matrix experiment. Proportions were arcsine square‐root transformed before analysis. *p* Values of random effects are based on likelihood ratio tests and *p* values of fixed effects on *F* tests with Satterthwaite's approximation

Source of variation	ndf for fixed effects	ddf for fixed effects	*F* for fixed effects/LR $\chi_{1}^{2}$ for random effects	*p*
Block			0.572	.449
*Hamiltonella* infection	1	13	18.958	<.001
Aphid clone (*Hamiltonella* infection)			12.214	<.001
Parasitoid line	5	65	2.186	.066
*Hamiltonella* infection × parasitoid line	5	65	1.784	.128
Aphid clone (*H*. inf.) × parasitoid line			1.674	.199

### 3.4 Parasitoid genotypes influence the outcome of cage experiments

**Figure 4:**
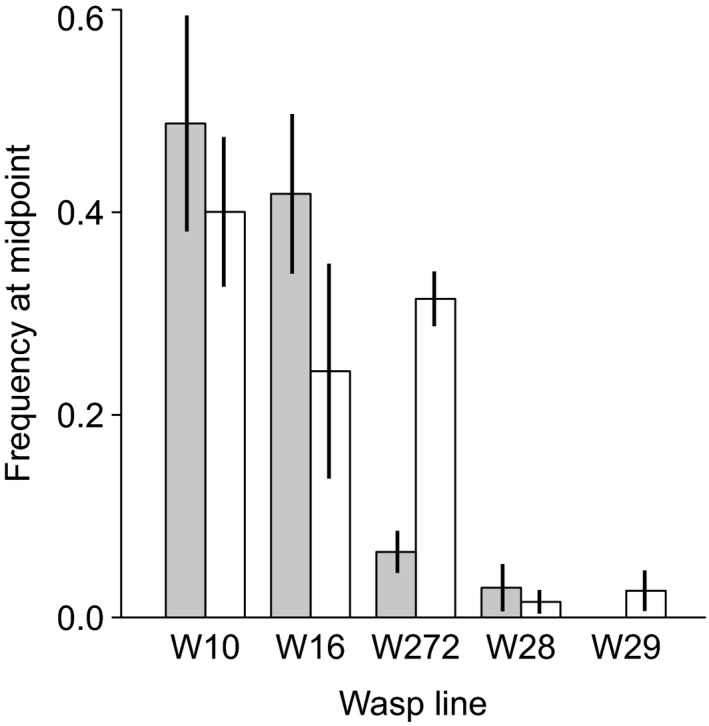
Bar plot showing the mean (± *SE*) relative frequencies in the midpoint samples for the five asexual lines of *Lysiphlebus fabarum* used in cages with asexual parasitoids, separately for cages in which parasitoids went extinct in the course of the experiment (gray bars, five cages), and for cages in which parasitoids either persisted until the end of the experiment (four cages) or even extirpated the aphids (one cage) (white bars)

In the 10 cages containing asexual parasitoids, we genotyped a subsample of wasps at the midpoint and, in those cages where parasitoids did not go extinct, also at the end of the experiment (four cages still had mummies at the last count, but only three yielded enough adult wasps for genotyping when the experiment was terminated, namely cages 4, 14, and 15). The relative frequencies of the five wasp lines at the midpoint corresponded well with their infectivities as estimated in the infection matrix experiment. The two least infective lines (W28 and W29) had nearly disappeared, the two lines that were most infective on unprotected aphids (W10 and W16) had increased strongly, and W272, the only line capable of parasitizing *H. defensa*‐protected aphids, had an intermediate mean frequency (Figure 4). At the end of the experiment, however, all cages still containing parasitoids had wasp populations consisting exclusively of line W272 (cage 4: *n *=* *36 wasps genotyped; cage 14: *n *=* *36; cage 15: *n *=* *8; all belonged to line W272). Only these wasps were able to persist when aphid populations became dominated by clones possessing *H. defensa*, and it appears that the speed with which line W272 reached a sufficient population size was decisive for whether parasitoid populations went extinct or not. This became evident when we compared the estimated midpoint frequencies of the five asexual lines between cages where parasitoids went extinct (five cages) and cages where parasitoids either persisted (four cages) or even extirpated the aphids (one cage) (Figure 4). The midpoint frequency of line W272 was significantly higher in the latter group (*t*_8_ = −7.3163, *p *<* *.001), while there was no significant difference in the frequencies of the other four lines (all *p *>* *.2).

## 4 DISCUSSION

This population cage experiment provided no evidence that the reproductive mode of parasitoids (sexual or asexual) influenced their effectiveness as biocontrol agents, independent of the genotypic diversity present in their host populations. However, our ability to detect any differences was limited because the predominant outcome was a failure of aphid control by both sexual and asexual parasitoids. Although the parasitoids established well in the cages and even seemed to have some initial effect on aphid densities, they have been sustained on only the *H. defensa*‐free aphids. This resulted in strong selection for aphid clones protected by *H. defensa*, which began to dominate within just a few weeks, such that the highly resistant aphid populations were no longer affected by parasitoids and escaped control in most cases. This can be seen as an example of eco‐evolutionary dynamics (Schoener, 2011). The fast selective change in host resistance altered the population dynamics, here in a way that is undesirable from a biocontrol perspective.

The rapid evolution of symbiont‐conferred host resistance in turn imposed selection on parasitoids. This could be seen in the treatments with asexual wasps, where it was possible to track the relative frequencies of the five asexual lines initially added. In the cages where parasitoids persisted to the end of the experiment, their populations were fixed for line W272; that is, the only line showing some ability to parasitize aphids protected by *H. defensa*. Interestingly, this line was not the most successful on unprotected aphids. Two other asexual lines were more infective than W272 on aphids without *H. defensa* (W10 and W16, see Figure 3), and these were indeed the lines that built up populations most rapidly in the initial phase of the experiment, whereas the two least infective lines did very poorly in all cages (Figure 4). It is unclear if or to what extent the five asexual lines actually competed for susceptible hosts, but in cages where the rapid increase of lines W10 and W16 was at the expense of line W272, the parasitoids went extinct before the end of the experiment. Only in cages where line W272 had achieved frequencies of 20%–40% by count 5 (midpoint sample) did we see the persistence of parasitoid populations or even the successful extirpation of aphids (cage 13). The selective change in the wasps' genotype frequencies was thus decisive for their population dynamics.

Sexual populations of parasitoids also have the capacity to adapt to the presence of defensive symbionts in their hosts. This has been demonstrated using experimental evolution (Dion et al., 2011; Rouchet & Vorburger, 2014), although in experiments that reduced the risk of parasitoid extinction by supplying fresh hosts on new plants every generation. Under the more realistic conditions of the present experiment, in which only host plants were replaced but aphid and parasitoid populations left to develop freely, counteradaptation of sexual *L. fabarum* was apparently not fast enough to prevent extinction when host populations became dominated by highly resistant aphids. It is likely that not all parasitoid mortality was due to the failure of developing in symbiont‐protected aphids. When aphid populations grow unchecked by natural enemies, as they mostly were in the later phase of the experiment, plant quality deteriorates and parasitized aphids may die from stress or starvation before the wasps can complete their development. Nevertheless, this reflects that sexual parasitoids did not adapt to the point that they could keep the increasingly resistant host populations in check. Whether the evolution of symbiont‐conferred resistance was simply too fast or whether the sexual parasitoids lacked the genetic variation required for rapid counteradaptation is difficult to tell. In hindsight, considering that the asexual parasitoids happened to comprise one line able to parasitize some of the *H. defensa*‐protected clones, the evolutionary potential of the asexual wasps was apparently higher than that of the sexual wasps, because they were completely unsuccessful on protected aphids in the infection matrix experiment (Figure 3). That said, we know from a different study that given enough time, increased infectivity on *H. defensa*‐protected aphids can be selected for in the same stock of sexual *L. fabarum* as was used here (Dennis, Patel, Oliver, & Vorburger, 2010).

There was one instructive exception to the general outcome in cages with sexual parasitoids. In cage 18, the parasitoids quickly increased to high density in the first weeks and then managed to persist and keep aphid densities low until the end of the experiment (Figure 1). This was a cage with low host genotypic diversity, and it contained the same three aphid clones as cage 13, where control by asexual parasitoids was successful and resulted in the extirpation of aphids. This suggests that A15, the *H. defensa*‐infected clone in these cages, may have been less well protected against parasitoids than the other clones harboring *H. defensa*. It is well known that different strains of *H. defensa* can vary in the strength of protection they provide (e.g., Cayetano, Rothacher, Simon, & Vorburger, 2015; Oliver et al., 2005). The results from the infection matrix support this hypothesis only partially, though. A15 was indeed one of the *H. defensa*‐infected clones that could be parasitized at least by wasp line W272, but it was not the most susceptible (Figure 3). Yet it has to be considered that the large size of the infection matrix restricted us to just three replicates per host‐parasitoid combination, which limits the reliability of these susceptibility estimates. The results from the cages with high host genotypic diversity, on the other hand, do support a limited resistance of clone A15. It was one of the *H. defensa*‐infected clones with low success and had nearly disappeared from all cages with high host genotypic diversity by the end of the experiment (Figure 2). In any case, the population dynamics observed in cages 13 and 18 show that it is sometimes possible for *L. fabarum* to keep *A. fabae* populations comprising symbiont‐protected clones in check, but generally, this was not the case with the aphids we used, which represented a haphazard and presumably representative sample from a natural population.

*Hamiltonella defensa* is a widespread endosymbiont of aphids (Henry, Maiden, Ferrari, & Godfray, 2015) and occurs in several economically important pest aphids (Zytynska & Weisser, 2016). Our observation that aphid control failed mostly, due to a rapid increase in symbiont‐protected aphids, is therefore discouraging for biological control with parasitoids. However, inoculative or inundative releases of parasitoids are generally considered successful strategies in real‐life situations and have become the method of choice for aphid control in greenhouses (Boivin et al., 2012; van Lenteren, 2012). This apparent discrepancy may be explained by the fact that we added a small inoculum of parasitoids to an already large aphid population, which may have prevented a more successful outcome. Suppliers of parasitoids for biological control emphasize that it is important to release parasitoids early, such that high parasitoid‐to‐aphid ratios can be reached at the onset of infestations (Neuville, Le Ralec, Outreman, & Jaloux, 2016; Van Driesche & Heinz, 2004). This can also be achieved with banker plant systems, that is, by growing an additional plant with nonpest aphids in the greenhouse to maintain generalist parasitoids that are then ready to meet the first colonizers of the pest aphids on the crop (Frank, 2010). Indeed, superparasitism (multiple ovipositions in the same host) as a result of high parasitoid densities may increase parasitoid success on symbiont‐protected aphids (Oliver et al., 2012), and when sufficiently numerous, parasitoids may also kill resistant aphids, either by stabbing them to death or disturbing them to the point of starvation (Hertäg, 2016). Expedient release strategies may thus mitigate the problem, but it is clear that parasitoid releases will often result in the rapid evolution of symbiont‐conferred resistance, as also demonstrated by Oliver et al. (2008) and Sanders et al. (2016). The present study suggests that more than their reproductive mode, the presence of genotypes able to overcome the resistance conferred by *H. defensa* is important for biocontrol success. Such genotypes can be found in natural populations (e.g., Vorburger & Rouchet, 2016), and parasitoid infectivity on *H. defensa*‐protected aphids can also be improved by selective breeding (Dion et al., 2011; Rouchet & Vorburger, 2014). Exploiting these opportunities may help to make biological control of pest aphids more effective.

## DATA ARCHIVING STATEMENT

Data available at Dryad Digital Repository: https://doi.org/10.5061/dryad.63124.
