# Supplementary material for: Rapid evolution of symbiont‐mediated resistance compromises biological control of aphids by parasitoids
Source: Evol Appl. 2017 Sep 3;11(2):220–30. doi: 10.1111/eva.12532 (PMC5775498; doi:10.1111/eva.12532)
Supplement: Supplementary file 1 [file EVA-11-220-s001.docx]

**SUPPLEMENTARY ONLINE MATERIAL**

Table S1. Collection information and genotypes at eight microsatellite loci (Coeur d'Acier et al., 2004) for the 15 clones of *Aphis fabae* used in this study.

| Clone | Lab. ID | Collection site | Latitude/longitude | Collection date | Host plant | Secondary symbiont | Used in low diversity cages nr.: |
| --- | --- | --- | --- | --- | --- | --- | --- |
| A1 | A12-1 | Steinmaur, CH | 47°30'N, 8°27'E | 27.05.2012 | *Chenopodium album* | *Hamiltonella defensa* | 12, 17 |
| A3 | A12-3 | Steinmaur, CH | 47°30'N, 8°27'E | 27.05.2012 | *Chenopodium album* | *-* | 15, 20 |
| A5 | A12-5 | Steinmaur, CH | 47°30'N, 8°27'E | 27.05.2012 | *Beta vulgaris* | *-* | 11, 16 |
| A7 | A12-7 | Steinmaur, CH | 47°30'N, 8°27'E | 02.06.2012 | *Beta vulgaris* | *-* | 14, 19 |
| A8 | A12-8 | Steinmaur, CH | 47°30'N, 8°27'E | 02.06.2012 | *Beta vulgaris* | *Regiella insecticola* | 12, 17 |
| A9 | A12-9 | Steinmaur, CH | 47°30'N, 8°27'E | 02.06.2012 | *Beta vulgaris* | *-* | 15, 20 |
| A10 | A12-10 | Steinmaur, CH | 47°30'N, 8°27'E | 02.06.2012 | *Beta vulgaris* | *Hamiltonella defensa* | 15, 20 |
| A12 | A12-12 | Steinmaur, CH | 47°30'N, 8°27'E | 02.06.2012 | *Beta vulgaris* | *Regiella insecticola* | 13, 18 |
| A13 | A12-13 | Steinmaur, CH | 47°30'N, 8°27'E | 02.06.2012 | *Beta vulgaris* | *-* | 12, 17 |
| A14 | A12-14 | Steinmaur, CH | 47°30'N, 8°27'E | 02.06.2012 | *Chenopodium album* | *Hamiltonella defensa* | 11, 16 |
| A15 | A12-15 | Dübendorf, CH | 47°24'N, 8°36'E | 07.06.2012 | *Chenopodium album* | *Hamiltonella defensa* | 13, 18 |
| A16 | A12-16 | Steinmaur, CH | 47°30'N, 8°27'E | 16.06.2012 | *Chenopodium album* | *-* | 11, 16 |
| A17 | A12-17 | Steinmaur, CH | 47°30'N, 8°27'E | 16.06.2012 | *Beta vulgaris* | *-* | 14, 19 |
| A20 | A12-20 | Steinmaur, CH | 47°30'N, 8°27'E | 17.06.2012 | *Chenopodium album* | *-* | 13, 18 |
| A204 | A06-204 | Steinmaur, CH | 47°30'N, 8°27'E | 10.06.2006 | *Chenopodium album* | *Hamiltonella defensa* | 14, 19 |

Table S1 (continued)

|  | Microsatellite locus | | | | | | | |
| --- | --- | --- | --- | --- | --- | --- | --- | --- |
| Clone | AF-48 | AF-50 | AF-82 | AF-85 | AF-86 | AF-181 | AF-F | AF-beta |
| A1 | 313 315 | 257 257 | 177 177 | 220 222 | 215 217 | 309 317 | 127 127 | 280 282 |
| A3 | 313 317 | 272 272 | 177 177 | 220 222 | 217 219 | 311 311 | 127 136 | 280 282 |
| A5 | 315 315 | 257 272 | 177 177 | 220 220 | 215 217 | 311 311 | 119 127 | 280 280 |
| A7 | 315 319 | 272 272 | 177 177 | 224 224 | 219 219 | 313 313 | 127 127 | 278 310 |
| A8 | 313 315 | 272 276 | 177 177 | 222 224 | 217 219 | 311 311 | 127 136 | 280 280 |
| A9 | 317 319 | 257 272 | 188 188 | 220 220 | 215 219 | 311 311 | 127 127 | 280 280 |
| A10 | 315 319 | 257 272 | 177 177 | 220 220 | 217 219 | 309 311 | 127 134 | 280 282 |
| A12 | 313 315 | 272 272 | 177 177 | 220 224 | 217 217 | 311 313 | 127 127 | 280 280 |
| A13 | 315 315 | 257 272 | 167 177 | 222 222 | 219 219 | 311 311 | 127 127 | 280 280 |
| A14 | 315 319 | 272 272 | 177 177 | 220 220 | 219 219 | 309 309 | 127 134 | 266 280 |
| A15 | 315 315 | 257 257 | 177 177 | 222 224 | 215 219 | 311 313 | 127 127 | 280 282 |
| A16 | 319 319 | 255 257 | 177 177 | 220 224 | 217 219 | 309 309 | 127 132 | 280 280 |
| A17 | 307 315 | 255 255 | 177 177 | 220 222 | 219 219 | 311 311 | 127 127 | 280 280 |
| A20 | 313 317 | 257 272 | 177 177 | 220 220 | 215 215 | 309 311 | 127 134 | 280 282 |
| A204 | 307 315 | 257 257 | 177 204 | 220 222 | 217 219 | 309 311 | 127 127 | 280 280 |

Table S2. Collection information for the nine samples of sexual *Lysiphlebus fabarum* that were mixed to form the sexual parasitoid population in this study.

| Sample ID | Collection site | Latitude/longitude | Collection date | Host aphid | Host plant |
| --- | --- | --- | --- | --- | --- |
| 12-51/56 | Lausanne, CH | 46°31'N, 6°38'E | 02.09.2012 | *Aphis hederae* | *Hedera helix* |
| 12-55 | Lausanne, CH | 46°31'N, 6°38'E | 02.09.2012 | *Aphis hederae* | *Hedera helix* |
| 12-62/83 | Fribourg, Ch | 46°48'N, 7°09'E | 12.09.2012 | *Aphis hederae* | *Hedera helix* |
| 12-69 | Fribourg, CH | 46°48'N, 7°09'E | 12.09.2012 | *Aphis hederae* | *Hedera helix* |
| 12-81 | Geneva, CH | 46°13'N, 6°09'E | 16.09.2012 | *Aphis hederae* | *Hedera helix* |
| 12-87/93 | Nyon, CH | 46°23'N, 6°14'E | 19.09.2012 | *Aphis hederae* | *Hedera helix* |
| 12-89 | Nyon, CH | 46°23'N, 6°14'E | 19.09.2012 | *Aphis hederae* | *Hedera helix* |
| 12-101 | Renens, CH | 46°32'N, 6°35'E | 19.09.2012 | *Aphis hederae* | *Hedera helix* |
| 12-154 | Thalwil, CH | 47°18'N, 8°34'E | 21.07.2012 | *Aphis hederae* | *Hedera helix* |

Table S3. Collection information and genotypes at ten microsatellite loci (Fauvergue et al., 2005; Sandrock et al., 2007) for the five asexual lines of *Lysiphlebus fabarum* used in this study.

| Line | Lab. ID | Collection site | Latitude/longitude | Collection date | Host aphid | Host plant |
| --- | --- | --- | --- | --- | --- | --- |
| W10 | IL12-10 | Regensberg, CH | 47°29'N, 8°26'E | 17.06.2012 | *Aphis hederae* | *Hedera helix* |
| W16 | IL12-16 | Zürich, CH | 47°22'N, 8°33'E | 21.06.2012 | *Aphis fabae cirsiiacanthoides* | *Cirsium arvense* |
| W28 | IL12-28 | Dübendorf, CH | 47°24'N, 8°36'E | 10.07.2012 | *Aphis hederae* | *Hedera helix* |
| W29 | IL12-29 | Wallisellen, CH | 47°25'N, 8°35'E | 17.06.2012 | *Aphis hederae* | *Hedera helix* |
| W272 | IL06-272 | Vezin, F | 48°07'N, 1°45'W | 14.06.2006 | *Aphis hederae* | *Hedera helix* |

Table S3 (continued)

|  | Microsatellite locus | | | | | | | | | |
| --- | --- | --- | --- | --- | --- | --- | --- | --- | --- | --- |
| Line | Lysi01 | Lysi03 | Lysi05 | Lysi06 | Lysi07 | Lysi08 | Lysi13 | Lysi15 | Lysi16 | Lysi5a12 |
| W10 | 079 079 | 165 165 | 112 112 | 195 203 | 183 183 | 099 105 | 121 121 | 107 107 | 135 135 | 176 176 |
| W16 | 079 079 | 167 167 | 110 110 | 197 201 | 183 183 | 092 094 | 121 125 | 103 103 | 117 117 | 176 176 |
| W28 | 079 079 | 167 169 | 112 112 | 195 195 | 183 183 | 101 101 | 121 125 | 105 105 | 123 123 | 178 178 |
| W29 | 079 079 | 161 169 | 110 112 | 195 197 | 183 183 | 088 101 | 121 125 | 109 109 | 125 125 | 178 178 |
| W272 | 079 079 | 165 167 | 112 112 | 195 199 | 183 183 | 094 107 | 123 125 | 103 103 | 117 117 | 176 176 |

**References**

Coeur d'Acier, A., M. Sembene, P. Audiot, and J. Y. Rasplus. 2004. Polymorphic microsatellites loci in the black Aphid, *Aphis fabae* Scopoli, 1763 (Hemiptera, Aphididae). *Molecular Ecology Notes* 4 (2):306-308.

Fauvergue, X., C. Tentelier, G. Genson, P. Audiot, T. Guillemaud, and R. J. Streiff. 2005. Microsatellite DNA markers for *Lysiphlebus testaceipes*. *Molecular Ecology Notes* 5 (1):109-111.

Sandrock, C., N. Frauenfelder, S. Von Burg, and C. Vorburger. 2007. Microsatellite DNA markers for the aphid parasitoid *Lysiphlebus fabarum* and their applicability to related species. *Molecular Ecology Notes* 7 (6):1080-1083.
